# Placental Mesenchymal Dysplasia: A Case Report

**DOI:** 10.1155/2012/202797

**Published:** 2012-08-05

**Authors:** Rachna Agarwal, Ritu Khatuja, Lipi Sharma, Alpana Singh

**Affiliations:** Department of Obstetrics & Gynaecology, University College of Medical Sciences and Guru Teg Bahadur Hospital, Delhi 110095, India

## Abstract

*Introduction*. A rare case of histologically proven placental mesenchymal dysplasia (PMD) with fetal omphalocele in a 22-year-old patient is reported. *Material and Methods*. Antenatal ultrasound of this patient showed hydropic placenta with a live fetus of 17 weeks period of gestation associated with omphalocele. Cordocentesis detected the diploid karyotype of the fetus. Patient, when prognosticated, choose to terminate the pregnancy in view of high incidence of fetal and placental anomalies. Subsequent histopathological examination of placenta established the diagnosis to be placental mesenchymal dysplasia. *Conclusion*. On clinical and ultrasonic grounds, suspicion of P.M.D. arises when hydropic placenta with a live fetus presents in second trimester of pregnancy. Cordocentesis can detect the diploid karyotype of the fetus in such cases. As this condition is prognostically better than triploid partial mole, continuation of pregnancy can sometimes be considered after through antenatal screening and patient counseling. However, a definite diagnosis of P.M.D. is made only on placental histology by absence of trophoblast hyperplasia and trophoblastic inclusions.

## 1. Introduction

Placental mesenchymal dysplasia (PMD) is an extremely rare histological condition in which there is massive hydrops of placental stem villi with absence of trophoblast hyperplasia and trophoblastic inclusions. Its reported incidence is 0.02% of all pregnancies [1]. The fetus is of diploid karyotype and the condition may be associated with Beckwith Weidmann syndrome in 25% of the cases [2]. We report a case of PMD at 17 weeks of gestation in a 22-year-old female with associated fetal omphalocele. The differentiating features from partial mole and pathological findings are also highlighted.

## 2. Case Report

**Figure 1:**
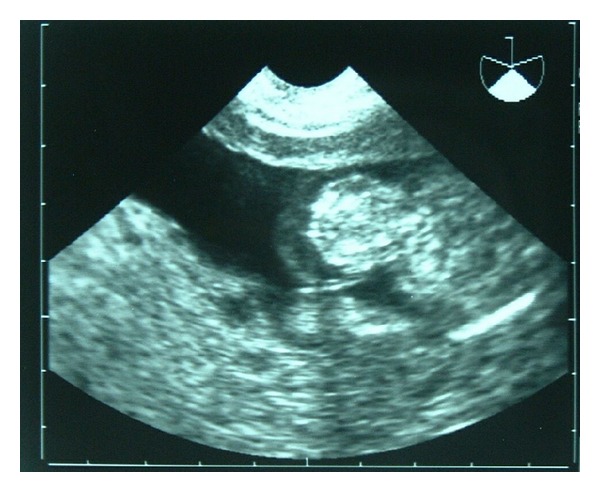
Ultrasound: fetus with omphalocele.

**Figure 2:**
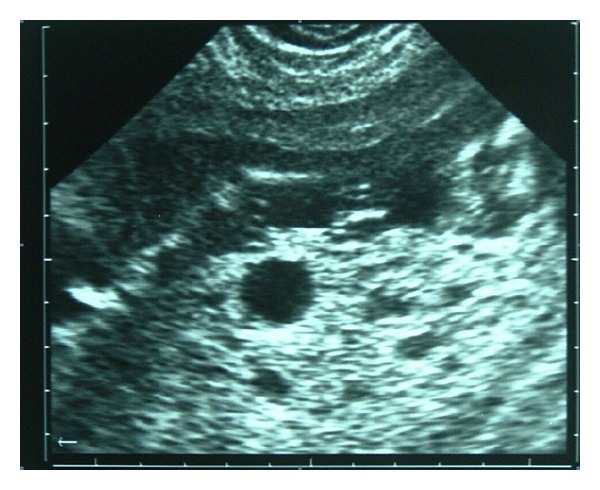
Ultrasound: Placentomegaly with multiple large anechoic areas. Is the entity partial mole?

Mrs. A, gravida 2 with previous one living issue, presented at 17 weeks gestation with spotting per vaginum for one week. Her obstetric and past history was unremarkable. On clinical examination, the fundal height corresponded to 22 weeks period of gestation. Ultrasound revealed a single live fetus corresponding to the period of gestation associated with omphalocele (Figure 1). Placenta was 40 mm thick and showed multiple large anechoic areas suggestive of partial mole (Figure 2). Serum *β*-human chorionic gonadotrophin (*β*HCG) was increased to 41,000 mIU/mL, and cordocentesis performed at this time revealed a diploid karyotype of the fetus. A presumptive diagnosis of PMD with omphalocele was made. When prognosticated in view of high incidence of fetal anomaly associated with this condition, patient opted for termination of pregnancy. She aborted after instillation of 800 ug of misoprostol intravaginally. The abortus was a female fetus of 250 gm with omphalocele and no other congenital anomaly was found on grossing. Placenta was 20 × 11 × 6 cm size, weighed 580 gm, and had multiple grape-like vesicular structures filled with serous fluid. Placental histopathology showed hydropic degeneration of villi with absence of trophoblast hyperplasia. This characteristic placenta and diploid karyotype of fetus were confirmative of placental mesenchymal dysplasia or “pseudopartial mole.” After termination, her serum *β*HCG was 3600 mIU/mL, which decreased to 440 mIU/mL at 90 days and returned to normal after 140 days. Postabortal followup was uneventful till 12 months. 

## 3. Discussion

PMD is a rare placental vascular malformation. Fetal omphalocele is another rare anomaly with an incidence of 1 : 3200 to 1 : 40000 [3]. Occurrence of PMD with isolated fetal omphalocele is extremely unusual. With a diploid fetus, PMD often poses diagnostic and management dilemma for the treating obstetrician.

The exact pathogenesis of PMD association with omphalocele is still unknown. Doubts have been raised about hydrops of placenta as a sequel to umbilical cord obstruction. Interestingly, female predominance has been seen in the literature. There may be a possible relationship between X chromosome and mesenchymal dysplasia [1].

In clinical practice, suspicion of PMD arises when a patient in second trimester undergoing triple screening shows elevated maternal serum *β*HCG and *α*-fetoprotein levels. Ultrasound performed at this stage may detect an enlarged placenta with numerous hypoechoic spaces and a viable fetus.

This condition is commonly confused with partial mole antenatally. The triploid fetus of partial mole may be differentiated on ultrasound by characteristic fetal malformations, such as syndactyly involving fingers 3-4 and toes 2-3, dysplastic cranial bones, and genital anomalies [4]. However, PMD is associated with either structurally normal fetus or Beckwith-Wiedemann syndrome (macrosomia, exomphalos, macroglossia, omphalocele, craniofacial features, and ear anomalies) in 25% of cases [5]. Additionally, the hypervascularity in the placenta may lead to fetal growth restriction or intrauterine fetal death and maternal hypertension [6].

A triploid karyotype in partial mole on cordocentesis helps differentiation between two entities. Sonographic findings of PMD can also resemble complete mole with cotwin or chorioangioma [7], and it is important to distinguish them as these conditions are associated with maternal and fetal complication. However, the confirmation of diagnosis is based on karyotyping and histopathological examination of placenta.

Postdelivery PMD is characterized by a large placenta with parenchymal vesicle and dilated vessels over its surface and weight usually greater than 95th percentile for gestation. Microscopic examination of placental tissue reveals massive hydrops of stem villi and characteristic absence of trophoblastic proliferation and villous trophoblastic inclusion. Terminal villi may be normal or mildly edematous. There may be chorioangiomatoid change of stem villous or aneurysmal dilatation of stem villous vessels [8, 9].

PMD is considered prognostically superior to partial mole as a diploid phenotypically normal fetus has better survival rates [8]. Patient can be prognosticated antenatally on suspicion of PMD after ultrasound screening and cordocentesis and counselled thereafter accordingly regarding further continuation of pregnancy.

This paper enlists the salient features of PMD for awareness among the ultrasonologists and obstetricians and stresses its identification as a distinct entity.
